# Organic–Inorganic Hybrid Nanoparticles for Enhancing Adhesion of 2K Polyurethane to Steel and Their Performance Optimization Using Response Surface Methodology

**DOI:** 10.3390/polym16192816

**Published:** 2024-10-04

**Authors:** Thu Thuy Duong, Manh Linh Le, Changhoon Lee, Juyoung Kim

**Affiliations:** 1 Nanocomposite Structure Polymer Laboratory, Department of Advanced Materials Engineering, Kangwon National University, Samcheok 25913, Republic of Koreaepoxy.plasma@gmail.com (C.L.); 2 VN-UK Institute for Research and Executive Education, The University of Danang, Danang 550000, Vietnam

**Keywords:** organic–inorganic hybrid, 2K polyurethane, alkoxysilane, adhesion strength, response surface methodology

## Abstract

Automakers are focusing on lightweight vehicles to address fuel economy and emission challenges and are using high-performance materials such as 2K PU-based joints as alternatives to cast iron, steel, and other metals. This study was conducted with the aim of expanding the application of 2K PU and enhancing its compatibility with steel substrates, which are commonly used in the automotive manufacturing industry, through the use of O-I hybrid nanoparticles containing alkoxysilane groups as additives in the 2K PU formulation. At the same time, the simplified process introduced and examined in this study demonstrates its feasibility for industrial-scale applications; the process offers notable advantages in reducing workload and curing time by eliminating cumbersome surface pretreatment steps before applying the 2K PU layer. Two types of commercial SB PU and EB PU were selected to study the mechanism by which O-I hybrid NPs enhance adhesion when integrated directly into the 2K PU formulation. We optimized various input parameters through practical work and modeling using the response surface method. These parameters included the amounts of AFAP precursor, APTES, and butylene glycol (BG) and the mixing ratio of O-I hybrid NPs in the formulations of two commercial PUs. The results show that O-I hybrid NPs significantly enhance adhesion, increasing performance on stainless surfaces by up to 2.35 times compared to pristine EB and SB PU. Notably, the SB PU’s performance can improve up to 2.5 times according to the RSM predictions, highlighting the substantial impact of O-I hybrid NPs.

## 1. Introduction

The outstanding properties of 2K PU, including high mechanical strength, superior chemical resistance, and strong adhesion, have resulted in its widespread application across various fields, such as packaging, furniture, construction, and automobile manufacturing [1,2,3,4]. In particular, the global automotive industry, in its efforts to reduce vehicle weight to achieve the goals of lowering CO_2_ emissions and conserving energy, has recently recognized that the application of 2K PU and lightweight materials is the most effective strategy [5]. 2K PU adhesives are a crucial component of ABJ (adhesively bonded joint) parts in automobiles; therefore, they must have the ability to bond effectively to a variety of substrates [6]. While some substrates exhibit excellent adhesion strength due to their similarities with 2K PU, others, particularly those made from metal, steel, and alloy materials, show poor adhesion as they are primarily composed of inorganic compounds [6]. Differences in compatibility, chemical reactivity, surface properties, and thermal expansion between organic 2K PU and inorganic materials hinder strong adhesion and the thermal stability of the joint [7,8]. Thus, developing an intermediary compound or additive is necessary to enhance the adhesion between 2K PU and metal substrates without increasing the overall workload or the total weight of the coating.

Our previous research successfully enhanced the compatibility of commercial 2K PU, including polyester polyol-based PU (SB PU) and polyether polyol-based PU (EB PU) (purchased from T&L Co., Yongin-si, Republic of Korea), with steel substrates through the application of O-I hybrid materials, achieving superior performance compared to pristine 2K PU [3]. Two different groups of O-I hybrid NPs (ATS and AGS) acted as adhesion promoter layers, coating the steel surface before the application of commercial 2K PU. Their unique structure, containing both inorganic segments made up of siloxane compounds and organic segments comprising urethane segments, ensured compatibility with both the steel surface and the PU layer [1,3]. However, as mentioned above, due to the practical needs and specific requirements of the machinery part processing and automotive industries, this process remains quite cumbersome as it requires increased overall workload and curing time [6,9,10]. Therefore, this study was conducted to further investigate the potential for the enhancement of the adhesion performance of O-I hybrid NPs when integrated into the commercial 2K PU formulation, known as SB and EB PU; the goal was to simplify the application process by eliminating the substrate surface pretreatment step.

A new process is proposed in this study. Instead of being pre-coated on the steel substrate, ATS was dispersed in butanediol, and a very small amount was used as an additive which was mixed with the resin component before being combined with the hardener solution to create 2K PU containing O-I hybrid NPs. The following input parameters that may affect adhesion performance were considered and investigated: the type of ATS, the amount of BG used to disperse ATS, and the mixing ratio of the dispersed ATS in BG to the components in the resin formulation of both commercial PU types. Notably, this approach has minimal impact on the commercial 2K PU formula, as the mixing ratio is very small (only about 0.24–1.19% of the total mass of PU). This ensures that the optimal ratio of isocyanate to hydroxyl groups (NCO/OH), as provided by the manufacturer, is essentially preserved.

The traditional method of single-factor control variable analysis was applied in the initial phase of this study. However, this method is both complex and time-consuming, posing limitations for large-scale manufacturing. Therefore, after successfully identifying the significant input parameter ranges that positively affect the adhesion performance of 2K PU in phase one, we simulated the adhesion performance using the central composite design (CCD) model, which is part of the response surface methodology (RSM), as the second phase. RSM is a statistical mathematical model first introduced in the 1950s; it has since been applied in various fields, particularly in chemical processes and manufacturing, due to its simplicity, cost-effectiveness, and time-saving advantages [11]. This method not only optimized the adhesion performance through mathematical modeling but also generalized the PU synthesis scenarios based on the three aforementioned factors. Due to the multi-step complexity of synthesizing 2K PU containing ATS, the application of RSM helps us confirm the optimal range of input parameters for the synthesis of 2K PU containing ATS particles. Thus, the experimental results were verified, and this study brought us closer to future research on the scaling up and industrialization of the model.

## 2. Materials and Methods

### 2.1. Chemicals

Isophorone diisocyanate, APTES, polyethylene glycol (PEG; Mw = 300 g/mol), dibutyltin dilaurate, ethanol, hydrochloric acid (HCl; ACS reagent, 37%), 1,4-BG, and MgSO_4_ were purchased from Sigma Aldrich (St. Louis, MO, USA) Chemical Co. Glycerol propoxylate (GP; Mw = 266 g/mol) was purchased from KPX Chemical Co. (Seoul, Republic of Korea) Two types of commercial 2K PU, polyester polyol-based PU (SB PU) and polyether polyol-based PU (EB PU), were purchased from T&L Co., Yongin-si, Republic of Korea.

### 2.2. Synthesis of AFAP

The synthesis of AFAP precursors was carried out using a three-step process reported in previous works [3,12,13,14]. The AFAP precursors were synthesized using GP (Mw = 266 g/mol) and PEG (Mw = 300 g/mol). This type of AFAP precursor demonstrated the highest adhesion performance compared to other AFAP precursors, as shown in our previous study [3].

### 2.3. Synthesis of O-I Hybrid NPs Dispersed in BG

Three types of O-I hybrid NPs were prepared for dispersion in various amounts of BG; the three types were named ATS (1-2), ATS (1-1), and ATS (2-1), where ATS (1-2) corresponded to the fact that the weight mixing ratio of AFAP to APTES was 1 to 2; ATS (1-1) and ATS (2-1) were similarly prepared. In part, AFAP (5 g) and different amounts of APTES (5 g, 10 g, and 2.5 g) were dissolved in 40 g of ethanol; then, an appropriate amount of HCl (0.1 M) was added as the catalyst for hydrolysis and condensation. The reaction was conducted at 60 °C for 48 h, allowing the ATS to be synthesized and dispersed completely in the ethanol (for the ingredient details, please see Table 1).

To prepare ATS dispersed in BG, the moisture in all the ATS solutions was removed using MgSO_4_ as a desiccant, followed by rotary evaporation to completely remove the by-products and ethanol [15,16]. During this step, the ATS was simultaneously dispersed in various amounts of BG (30 g, 40 g, and 50 g). These types of O-I hybrid NPs were named ATS (1-2)–BG (30), ATS (1-1)–BG(30), ATS (2-1)–BG(30), ATS (1-2)–BG(40), and ATS (1-2)–BG(50), where ATS (1-2) denoted O-I hybrid NPs formed by AFAP and APTES at a mixing ratio of 1:2, and BG(30) indicated that 30 g of BG replaced ethanol to become the solvent of ATS (Table 2).

### 2.4. Preparation of SB/EB PUs Containing O-I Hybrid NPs

Like other commercial 2K PU products, SB and EB PU are composed of two parts: resin and hardener; the ingredients are specified in Table 3 (as provided by T&L Co., Yongin-si, Republic of Korea). The pristine SB and EB PU were considered as the reference PU. For the SB and EB PU containing O-I hybrid NPs, ATS–BG was used as an additive component in the resin part at nominal loadings of 1 wt%, 3 wt%, and 5 wt% of the SB and EB polyol mass, as described in Table 4. The adhesion performance of the PU samples containing O-I hybrid NPs was compared with the PU samples without O-I hybrid NPs using shear strength tests.

### 2.5. Multiparameter Experimental Design Model

The optimization process was conducted using the RSM with the Design Expert software. This process involved several steps, including estimating the coefficients, predicting the responses, and assessing the acceptability of the developed model. The response is represented by Equation (1):

(1) $$y=\beta_{0}+\sum_{j=1}^{k}\beta_{j}x_{j}+\sum_{i<j}\sum \beta_{ij}x_{i}x_{j}+\sum_{j=1}^{k}\beta_{jj}x_{j}^{2}+\epsilon$$

where y is the predicted response, β_0_ is the constant coefficient, β_j_ is the linear coefficient, β_ij_ is the interaction coefficient, β_jj_ is the pure second-order or quadratic effect, x is the design factor, and ∈ is an experimental/residual error.

The coded and actual levels of the independent variables are given in Table 5. The CCD model was selected, and the total number of experiments was 30, including 5 central point experiments. The coded and actual levels of the independent variables, including the amounts of AFAP (g), APTES (g), and BG (g), and the mass mixing ratio of ATS to optimize the adhesion strength of SB PU in the response are given in Table 4. A series of experiments designed based on the CCD model is presented in Table S1 (Supplementary Materials).

### 2.6. Characteristics

The microstructure analysis used field-emission scanning electron microscopy (FE-SEM; JEOL JSM-6701F/X-Max, Peabody, MA, USA) with energy-dispersive X-ray spectroscopy (EDS). After being subjected to the shear strength test, the pristine SB PU sample and the SB PU sample containing O-I hybrid particles were cut into small, 1 cm × 1 cm pieces to analyze the surface morphology and the failure mode of the PU layer.

A dynamic light scattering analyzer (DLS, Zetasizer Nano ZS, Malvern Instruments, Malvern, UK) was used to determine the particles size of different O-I hybrid solutions. All of the ATS samples in ethanol were diluted approximately 200 times with ethanol, then filtered using a syringe filter (0.2 micrometer pore size) before being measured using the DLS.

^29^Si-NMR (Avance III-500, Bruker, Leipzig, Germany) measurement was used to confirm the degree of polymerization of the different O-I hybrid NPs.

The moisture content of the samples was measured using a Karl Fischer titrator (SI Analytics, Mainz, Germany).

The FT-IR spectra of the urethane elastomers were obtained using a Vertex 70v spectrophotometer (Bruker Optic GmbH, Ettlingen, Germany). The prepared PU mixture was coated onto a glass substrate with an approximate thickness of less than 500 μm. It was then dried at room temperature for 24 h before being measured using ATR-FTIR. The scanning range was from 500 to 4000 cm^−1^. The carbonyl hydrogen bonding index (R) was derived from the intensities of the carbonyl stretching vibrations of the free (A_free_) and hydrogen-bonded (A_bonded_) groups, which are found from 1650 cm^−1^ to 1760 cm^−1^, respectively. The R index was computed using Equation (2).

(2) $$R=\frac{A_{bonded}}{A_{free}}$$

The degree of the phase separation (DPS) and the degree of the phase mixing (DPM) were obtained using Equations (3) and (4).

(3) $$\mathrm{DPS}=\frac{R}{R+1}$$

DPM = 1 − DPS (4)

### 2.7. Adhesion Strength Test

**Figure 1:**
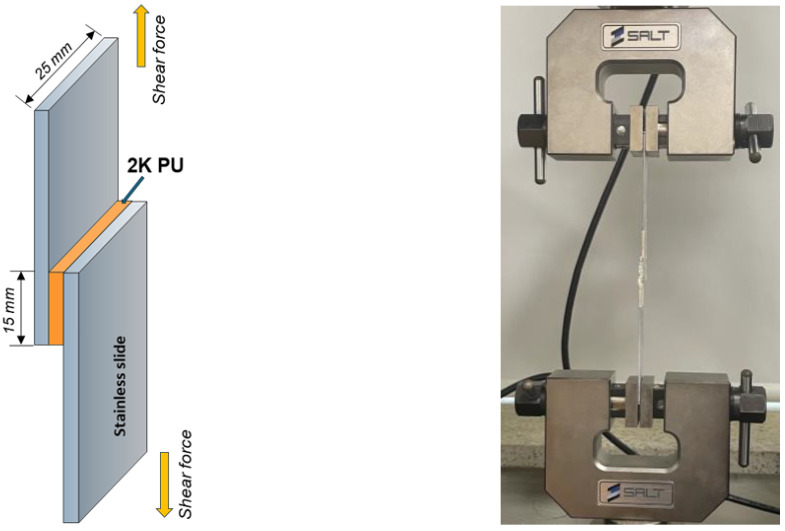
Shear strength testing for adhesion measurement of 2K PU.

The adhesion strength of the PU containing O-I hybrid NPs on a metal substrate was determined using the shear strength test method, as per the ASTM D-1002 standard. The metal specimens used in this test were standardized with a width of 25 mm, an overlap of 15 mm, a thickness of 1.5 mm, and an overall length of 100 mm (CR340, POSCO, Pohang, Republic of Korea). The PU solution was coated onto the metal specimens, as shown in Figure 1, then tested at a speed of 1.3 mm/min and a load cell of 1 ton. The measurements were repeated at least three times for each PU sample. All the metal slides used for this test had the same roughness, with an average surface of 1.339 μm, as analyzed using a stylus profilometer (VK-X1050, KEYENCE, Itasca, IL, USA).

## 3. Results and Discussion

### 3.1. Characteristic of O-I Hybrid NPs

**Figure 2:**
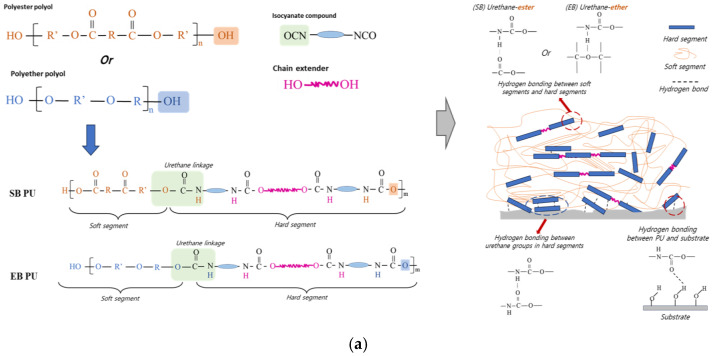

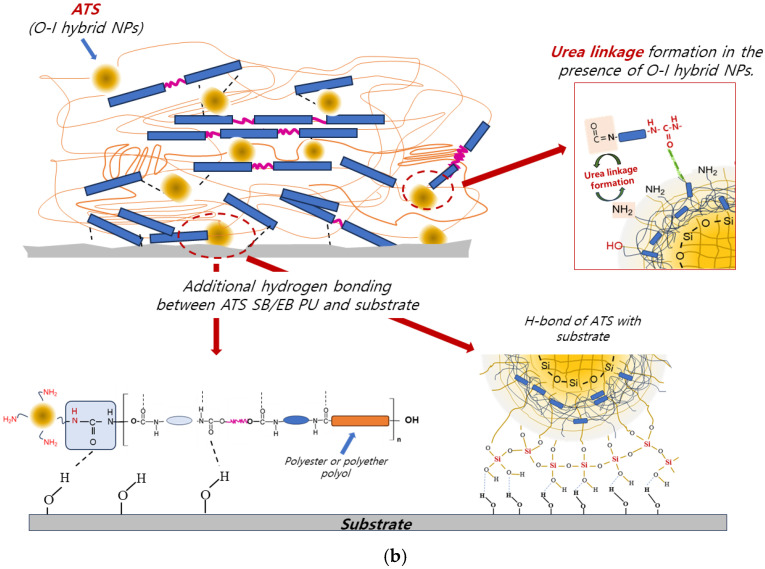
Schematic presentation (**a**) commercial SB/EB PU formation and (**b**) the mechanism of the adhesion strength improvement of ATS O-I hybrid NPs for SB/EB PU.

The formation of pristine SB/EB PU and the possible interaction mechanisms between the segments in the PU and the substrate are shown in Figure 2a. In the case of the dispersion of ATS in SB/EB PU, it is expected that by ensuring a highly ordered arrangement of hard and soft segments within the PU layers and increasing the density of the hydrogen bonds, the adhesion performance will be enhanced (Figure 2b). ATS can react with inorganic materials due to the contained siloxane groups and has compatibility with organic materials due to the amphiphilic organic segments on the surface (Figure 2b) [12]. In particular, the amphiphilic urethane segments from the AFAP precursor, which is used to synthesize ATS, are also compatible with the urethane segments in 2K PU. The amine groups in ATS react with isocyanate groups to form urea linkages (Figure 2b), which were proven to contribute to the increased adhesion of 2K PU through urea linkage-based hydrogen bonding [17,18]. At the same time, ATS enhances hydrogen bonding between the siloxane groups on the ATS steel surface (Figure 2b). All these factors contribute to the increase in the overall adhesion performance of 2K PU by enhancing the density and abundance of hydrogen bonding between 2K PU and the steel substrate [3,19,20].

Ethanol and moisture were demonstrated to have a negative impact on the adhesion performance of PU on the substrate; therefore, we proposed removing them from the ATS and then redispersing the ATS in BG [15,16]. Table 6 presents the particle size of the synthesized O-I hybrid NPs before and after the solvent exchange process (ATS and ATS–BG); the size ranged between 11.66 nm and 40.41 nm. Overall, the particle size of the ATS samples varied depending on the mass ratio of AFAP and APTES, and it increased after the ethanol was evaporated and replaced with BG as the solvent. The mixing ratio between AFAP and APTES affects the particle size of ATS; this is believed to be due to the significant difference in the molecular sizes of the two types. APTES is a relatively small silane molecule, while AFAP is a type of hybrid organic–inorganic polymer with a more complex structure that can create larger network structures [21]. After being redispersed in different amounts of background (BG), the particle size of ATS also showed differences in the following order: ATS–BG (50 g) < ATS–BG (40 g) < ATS–BG (30 g). This is because BG has a higher viscosity compared to ethanol, which limits the mobility of the NPs and leads to larger particle sizes. This suggests that the larger the amount of BG used, the smaller the particle size of the ATS–BG mixture.

**Figure 3:**
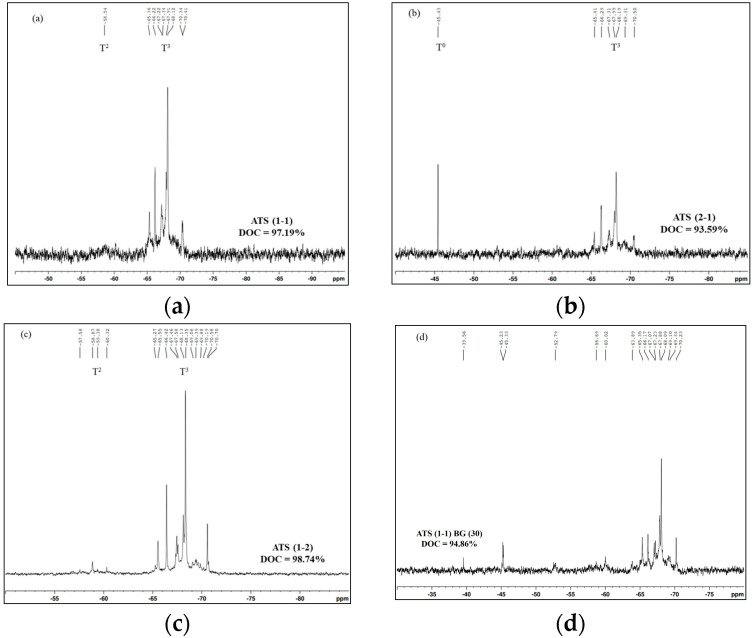

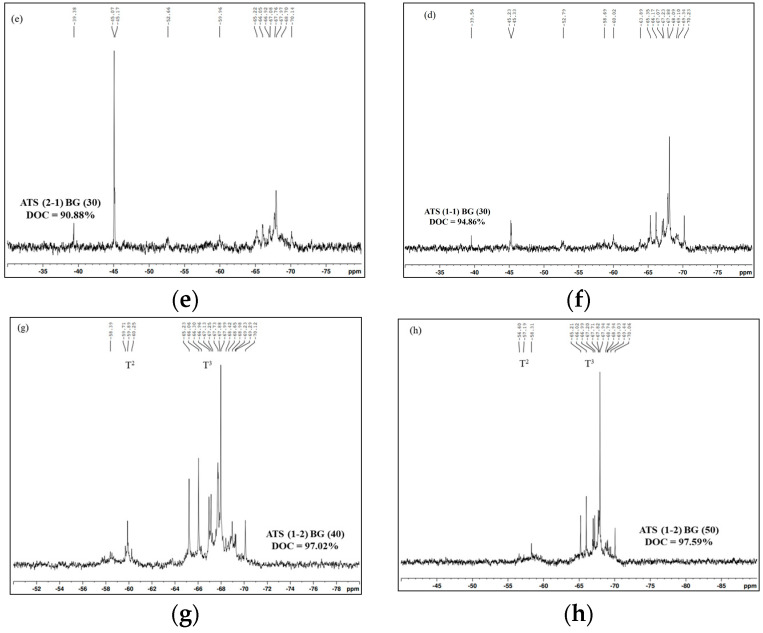
^29^Si-NMR spectra of (**a**) ATS (1-1), (**b**) ATS (2-1), (**c**) ATS (1-2), (**d**) ATS (1-1)–BG (30), (**e**) ATS (2-1)–BG (30), (**f**) ATS (1-2)–BG(30), (**g**) ATS (1-2)–BG(40), (**h**) ATS (1-2)–BG(50).

^29^Si-NMR measurement was used to analyze the degree of condensation (DOC) of the alkoxysilane compounds after the hydrolysis–condensation reaction (Equation (5)) [22]. The DOC refers to the level of crosslinking or condensation reactions that have occurred in the alkoxysilane compound to form a complex, high-molecular-weight compound, resulting in a more rigid and robust material. This can clarify the reasons for the different adhesion performances of the SB/EB PU samples in the next section, as higher levels of crosslinking have been demonstrated to provide greater adhesion strength [23]. Figure 3 and Table 6 illustrate that all the ATS samples exhibited DOC values exceeding 90%, indicating successful condensation bonding of the alkoxysilane groups to form polysiloxane compounds. In particular, O-I hybrid particles containing ATS (1-2) demonstrated higher DOC values even when dispersed in ethanol or redispersed in BG compared to the other O-I hybrid NPs containing ATS (2-1) and ATS (1-1). Despite a slight decrease in DOC values after solvent exchange (ranging only from 1.7 to 2.9%), this can be attributed to the viscosity difference between BG and ethanol. Nevertheless, this compromise is unavoidable as the goal of this process is to completely remove ethanol and moisture from the ATS solution in order to avoid the negative impacts they may have on the adhesion performance of PU.

(5) $$DOC=\frac{T^{1}+{2T}^{2}+{3T}^{3}}{3(T^{0}+T^{1}+T^{2}+T^{3})}\times 100$$

### 3.2. Adhesion Performance

**Figure 4:**
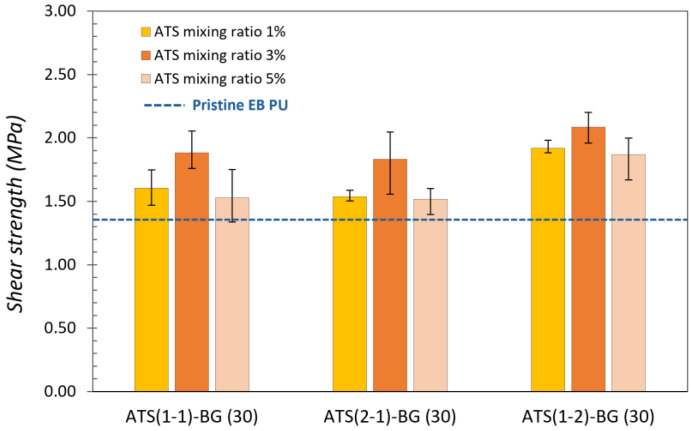
Adhesion strength of pristine EB PU and novel PU containing ATS.

**Figure 5:**
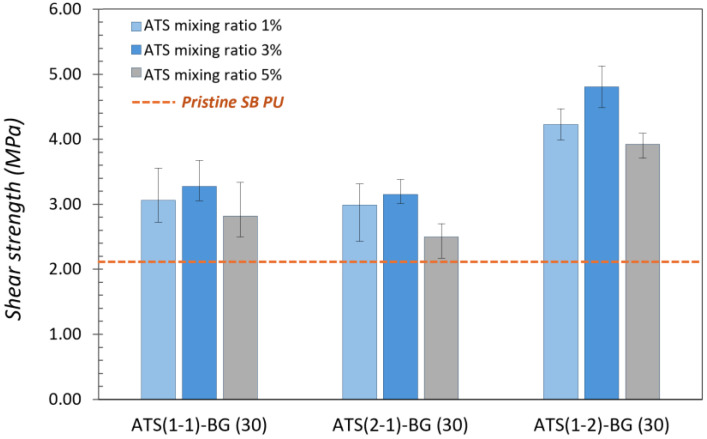
Adhesion strength of pristine SB PU and novel PU containing ATS.

The adhesion performances of the pristine EB and SB PU samples and nine types of each EB and SB PU containing O-I hybrid particles with mixing ratios ranging from 1% and 3% to 5% are illustrated in Figure 4 and Figure 5. The dashed lines represent the adhesion strength of the pristine EB and SB PU as a reference value, and the values are denoted as columns indicating the adhesion performance of the modified PUs. The adhesion performances of the pristine EB and SB PU were determined at 1.37 ± 0.21 MPa and 2.06 ± 0.35 MPa, respectively.

All the modified PU samples containing ATS–BG showed better performance than the pristine EB and SB PU. The highest adhesion strength was observed in EB and SB PU containing ATS (1-2)–BG (30)–3 wt%; the adhesion strengths were 2.09 ± 0.13 MPa and 4.86 ± 0.35 MPa, indicating that a higher content of ATS (1-2) with higher molecular weight species content and DOC can lead to better adhesion strength. Adding too few or too many O-I hybrid NPs can impact the adhesion performance of PU. Small amounts of O-I hybrid NPs may lead to only a slight increase in the density of the hydroxyl bonding. Conversely, adding too much of these NPs can increase the BG content in PU, upsetting the balance between the NCO and OH groups in the PU formulation, resulting in a decrease in adhesion performance. The NCO/OH value has been demonstrated to be an important parameter in the adhesion performance of PU [24,25].

**Figure 6:**
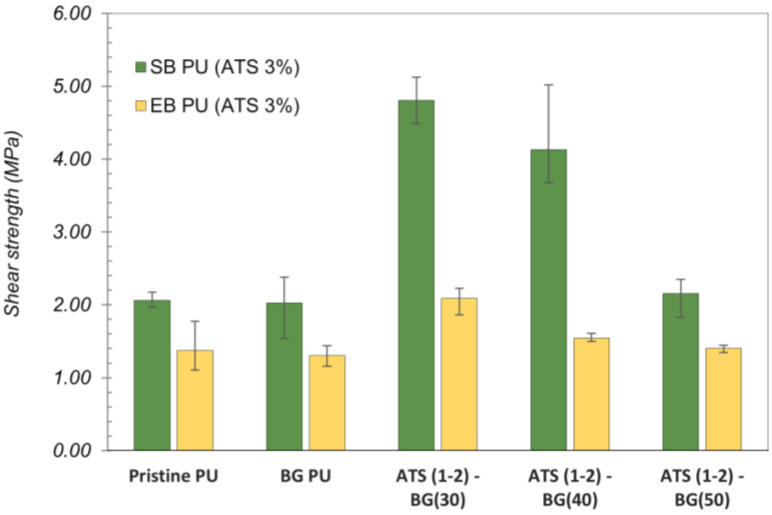
Adhesion strength (MPa) of SB PU and EB PU containing ATS dispersed in different amounts of BG.

To investigate the effect of dispersion on adhesion strength, the same type of ATS (1-2) was dispersed in different amounts of BG—30, 40, and 50 g—before being mixed with polyols at a fixed mass ratio of 3% to form modified EB and SB PU. Notably, an attempt was made to disperse ATS in a smaller amount of BG; however, the resulting mixture exhibited excessively high viscosity, preventing it from dispersing well in 2K PU. A total of four samples of PU containing O-I hybrid NPs of each type of SB or EB PU were then subjected to shear strength testing, and the results are shown in Figure 6. The highest adhesion strength was observed in both modified SB and EB PU when using ATS (1-2)–BG (30), compared to the formulations with higher quantities of BG (40 g and 50 g) for ATS dispersion. The adhesion strengths achieved were 4.84 ± 0.26 Mpa for the SB PU sample and 2.09 ± 0.17 Mpa for the EB PU sample. Specifically, for EB PU, the adhesion performance was 1.55 ± 0.06 MPa with the sample using ATS (1-2)–BG(40) and 1.40 ± 0.05 MPa with the sample using ATS (1-2)–BG(50), whereas for SB PU, the corresponding values were 4.13 ± 0.76 MPa and 2.16 ± 0.29 MPa. This could be attributed to variations in the content of O-I hybrid NPs within the ATS–BG mixture used to form the PU. When ATS is dispersed in a higher quantity of solvent BG, the weight distribution of ATS within the mixture decreases while the BG content increases. This results in an insufficient density of O-I hybrid NPs within the PU matrix while the higher amount of BG leads to a reduction in the NCO/OH ratio [24].

### 3.3. FT-IR

**Figure 7:**
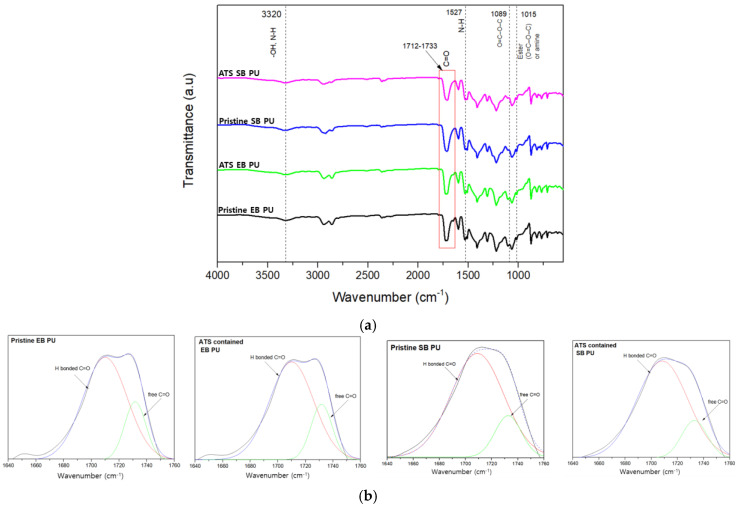
FT-IR spectra of (**a**) pristine EB and SB PU; SB PU and EB PU containing ATS (1 −2)–BG (30)–3 wt%; (**b**) absorption intensity of hydrogen-bonded C=O (red) and free C=O (green) fitted using Gaussian multipeak fitting.

The FT-IR spectroscopy experiments were conducted over the wavelength range of 500–4000 cm^−1^ and focused on three principal regions: -CH stretching (2700–2950 cm^−1^), -NH stretching (3500–3300 ^−1^), and C=O stretching (1640–1760 cm^−1^). The FTIR spectra of the two pristine PU samples and their corresponding ATS (1-2)–BG(30) 3% containing the PU forms are shown in Figure 7a. It was noted that in the bands attributed to N=C=O-, there were free groups indicating or confirming the complete reaction of urethane that formed in the pristine SB/EB PU group and the SB/EB PU group containing ATS. The absorption band at 3323 cm^−1^ corresponds to NH stretching. The sharp peaks at 2859 cm^−1^ and 2946 cm^−1^ are associated with −CH_2_ stretching. The group of NH vibrations is identified by the bands at 1527 cm^−1^.

PU can form various types of hydrogen bonds due to the presence of N-H donor groups and C=O acceptor groups within the urethane linkage. These bands are widely used to characterize the state of hydrogen bonding. It is well known that in hydrogen-bonded urethanes, N-H and C=O bands appear at lower wave numbers than the bands that appear in urethanes free from hydrogen bonding [26,27,28]. The band at 1710 cm^−1^ is assigned to the hydrogen bonding between the N-H and C=O groups in the hard segment and the ester or ester–oxygen groups of the soft segments of the urethane linkage. The band at 1733 cm^−1^ belongs to non-hydrogen-bonded carbonyl groups (Figure 7b). The curve fitting results are listed in Table 7. With the addition of ATS, the carbonyl hydrogen bonding index, R, increases. Specifically, R is defined as the ratio of the amount of carbonyl groups connected by the hydrogen bond (1710 cm^−1^) to the amount of free carbonyl groups (1733 cm^−1^). Moreover, though an increase in the phase separation degree, DPS, is observed, the degree of phase mixing, DPM, decreases. This means that, in fact, the urethane hard-phase domains, as presented in the microstructure, are increasing in size. Additionally, an increase in the R index indicates an increase in the density of hydrogen bonding in the 2K PU layer, which has been shown to lead to improved adhesion of the 2K PU layer to the metal substrate [19,20].

### 3.4. Morphology SB PU containing ATS

**Figure 8:**
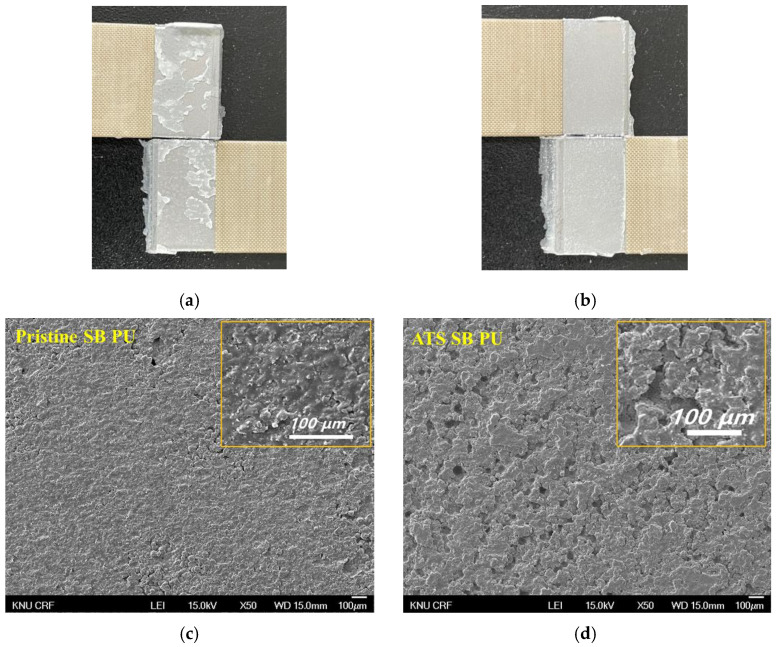

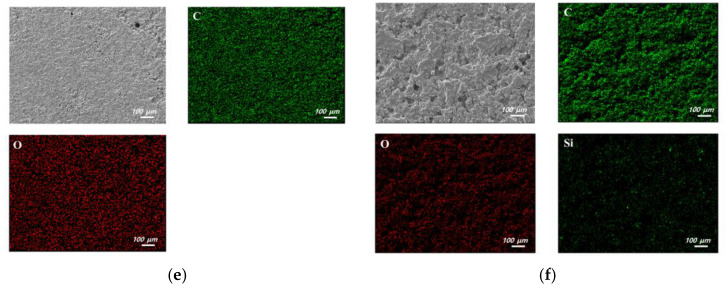
FE-SEM image and EDS analyses of detached surface of both pristine SB PU (**a**,**c**,**e**) and modified SB PU containing ATS (1-2)–BG(30)–3 wt% (**b**,**d**,**f**).

The morphology of PU surfaces can be used to confirm the presence of O-I hybrid NPs in modified PU. This analysis is carried out by examining the surface of the PU after it has been detached from the metal substrate in a shear strength test. SB PU containing ATS (1-2)–BG(30)–3 wt% was selected for a comparison with the pristine SB PU due to its superior adhesion performance, as shown in Figure 8. After detachment, both types of PU, the pristine SB PU and the SB PU containing ATS, exhibited a rough surface. However, the surface of the SB PU containing O-I hybrid NPs was rougher, with many small holes after detachment from the metal substrate (right side), whereas the pristine PU had a relatively smooth and uniform texture. This suggests that a certain degree of cohesive failure was likely to have occurred, as evidenced by the adhesive layer remaining on both surfaces due to the strong adhesion force between the adhesive layer and the steel surface. The EDS analysis results showed that the O-I hybrid NPs were present in the modified SB PU sample, with Si atoms properly distributed throughout the polymer matrix, resulting in a homogeneous and uniform microstructure.

### 3.5. Optimization Adhesion Strength

#### 3.5.1. Development of Regression Model Equation and Optimization

The quadratic model was fitted to the obtained responses, and analysis of variance (ANOVA) was performed. The coefficient of determination (R^2^), adjusted R^2^, coefficient of variation (CV), lack of fit, etc., were considered for the evaluation of the model’s significance. A *p*-value less than 0.05 indicated that the model terms were significant, and values greater than 0.10 indicated that the model terms were not significant. As shown in Table 7, the probability values for the terms A, B, C, AB, BC, B^2^, C^2^, and D^2^ are less than 0.05; thus, they are significant model terms. The lack of fit F-value of 1.71 implies that the lack of fit is not significant relative to the pure error, which indicates that the model fits well with the experimental data.

The following equations express the overall predictive model in terms of the variables:

Adhesion strength (Mpa) 
−5.69973 + 0.113088 × **AFAP** + 1.3691 × **APTES** + 0.158998 × **BG** + 0.349081 × **Mixing mass ratio**
+ 0.062894 × **AFAP** × **APTES** + 0.007095 × **AFAP** × **BG** + 0.011137 × **APTES** × **BG** − 0.008235 × **AFAP** × **Mixing mass ratio** + 0.018290 × **APTES** × **Mixing mass ratio** − 0.000388 × **BG** × **Mixing mass ratio**

−0.115125 × **AFAP^2^** − 0.104397 × **APTES^2^** − 0.004112 × **BG^2^** − 0.07092 × **[Mixing mass ratio]^2^** (6)

The coefficient of determination (R^2^) is another measure used to evaluate the adequacy of a model. As shown in Table 8, the difference between the adjusted R-squared (0.8583) and predicted R-squared values (0.6597 is <0.2 and the model’s adequate precision is 10.2524 (which is >4)) also indicates that the model is adequate.

**Figure 9:**
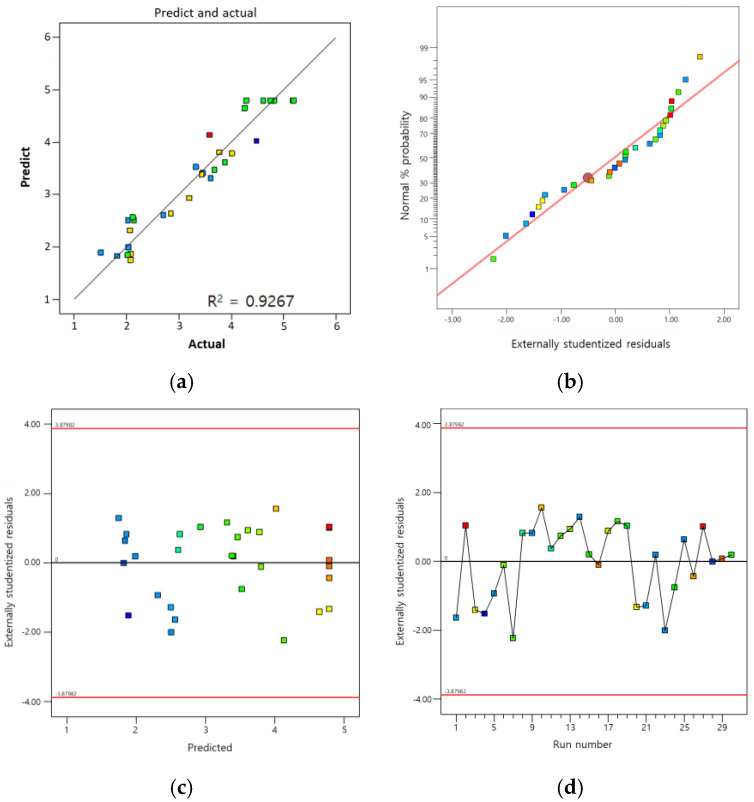
(**a**) Relationship between actual and predicted values using CCD/RSM model for SB PU containing ATS; (**b**) normal plot of residuals of adhesion strength data; (**c**) the plot of residuals vs. predicted response of adhesion strength data; (**d**) the plot of residuals vs. run of adhesion strength data.

Figure 9a shows the comparison between the actual response values as obtained from the experimental runs and the predicted response values based on the quadratic model equation for the adhesion strength of SB PU. From this graph, it can be clearly seen that the points lie close to the actual values, which shows that the predicted values are in good agreement with the actual values, with an R^2^ of 0.9282. In a predictive model, high accuracy is often indicated by an R^2^ value approaching 1.0. In this case, with four selected variables and 30 generated data points, this value is acceptable. Some samples were prepared then compared with the maximized adhesion strength predicted by the theoretical model, with the following conditions: 4.36 g of AFAP, 10.25 g of APTES, 37.16 g of BG, and a mixing ratio of 3.6% ATS (1-2)–BG(30). Although the latter value differs from the ratio observed in phase 1 when varying the individual parameters to find the optimal conditions for SB PU, it still falls within the investigated range; however, it represents a point not directly surveyed. Specifically, the AFAP to APTES ratio in traditional testing is 1:2, whereas this value is 1:2.35. The investigated ranges for BG were 30, 40, and 50 g, with the optimal condition being 30 g, while the predicted model value was 37.15 g; thus, it remained within the investigated range. Similarly, the mixing ratio of ATS (1-2) to BG (30) for 2K PU was comparable. This indicates that the model effectively generalized all the cases within the investigated range.

Furthermore, Figure 9b represents the fact that the errors are normally distributed, as the normal probability plot of the residuals shows that the errors fall in a straight line. The assumption of constant variance is satisfied by the predicted model, as the random scatter plot depicted in Figure 9c shows the distribution of residuals in a structureless form. Moreover, Figure 9d shows the random scatter of the residuals across the mean value of the residuals, which indicates that the residuals are independent of the run order.

#### 3.5.2. Three-Dimensional Surface Plots and the Effects of Variables on the Adhesion Strength

**Figure 10:**
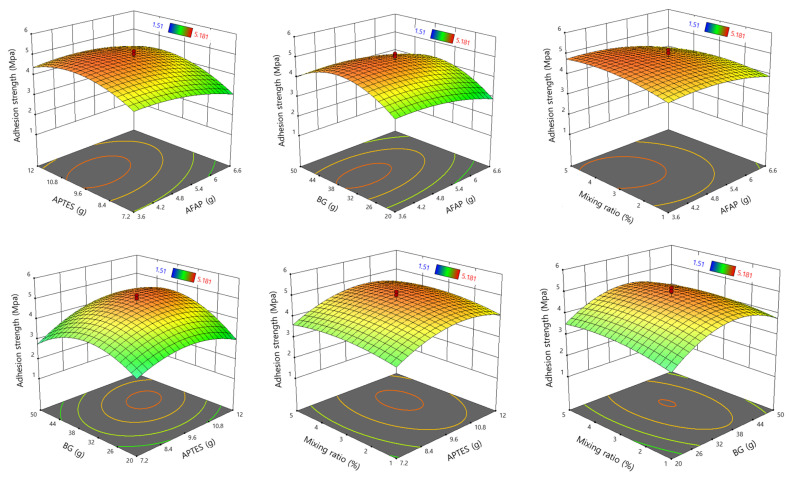
Three-dimensional plots for the simultaneous effect of 4 variations on adhesion strength of SB PU containing ATS.

To analyze the combined effect of the factors on the adhesion strength, the regression equation was graphically represented using 3D surface plots. The four factors create six combinations of pairwise interactions, as illustrated in Figure 10. The 3D surface graph shows the response/adhesion strength as a function of two independent variables while keeping the other two variables fixed. The curvature of the 3D graph illustrates the degree of influence of each factor on the adhesion performance. The different color codes of the 3D plots symbolize the values of the power density, as shown by the surface. These graphs show that each pair of variations influences the adhesion performance of SB PU.

#### 3.5.3. Confirmation Model

**Figure 11:**
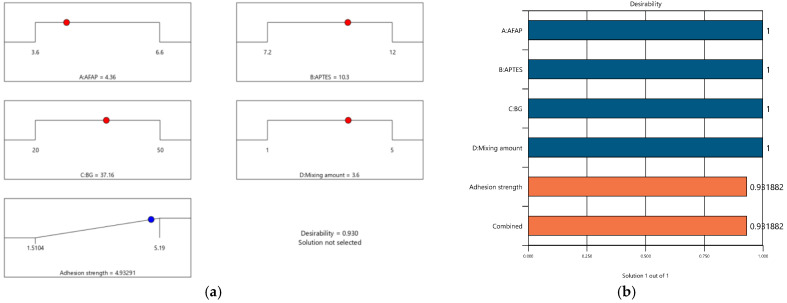
(**a**) Ramp function graph of desirability and (**b**) bar graph of desirability.

Figure 11a,b is a ramp plot and bar graph depicting the desirability of the various input and output qualities utilized in the present study. The optimization technique resulted in a cumulative desirability of 0.9318. At this level of desirability, the trade-off analysis determined the optimal amount of AFAP to be 4.36 (g), the amount of APTES to be 10.25 (g), the amount of BG to be 37.16 (g), and the mixing ratio to be 3.6%. At these optimal control parameters, the optimal adhesion strength of 4.94 Mpa was attained (Table 9).

The vital part of the experimental study involved the determination of the optimum process conditions required to obtain maximum adhesion strength. To confirm the results obtained from the Design Expert optimization method, the software’s predicted values were tested five times using experimental techniques. The optimized adhesion strengths predicted from the model and confirmed by experiment were 4.93 MPa and 4.88 ± 0.417 (MPa). The percentage of error between the predicted value and the experimental data was 1.2%; this was consistent with the calculated R^2^ value obtained from ANOVA. This consistency between the predicted and experimental results demonstrates the model’s reliability and its ability to accurately forecast the maximum adhesion strength of PU containing ATS (1-2)–BG(30). The details of the optimization conditions are shown in Table 10.

## 4. Conclusions

This work focuses on improving the adhesion performance of two types of commercial 2K PU (SB and EB PU) by incorporating glycol-dispersed O-I hybrid NPs. Using a small amount of ATS (1-2)–BG(30), specifically 3%, ensures that the mass of the adhesive layer does not change significantly, while the adhesion performance increases by 1.53 times and 2.35 times for EB PU and SB PU, respectively. Our study once again demonstrates that O-I hybrid NPs serve as nanoscopic fillers or crosslinking units in the 2K PU formulation and that they also provide high polarity functional groups and form more hydrogen bonds with the OH groups on the surface of the metal substrates, thereby significantly enhancing the adhesion strength between the PU and metal substrates. AFAP, APTES, BG, and the mixing ratio of ATS were identified as four crucial factors that can affect the adhesion strength of the modified 2K PU and were selected to design an optimized model for enhancing the adhesion performance of SB PU containing O-I hybrid NPs using RSM. The theoretical model demonstrated a strong correlation with real-world data, with an R-squared value of 0.928. The highest adhesion performance value from the model was 4.94 MPa, indicating that the adhesion performance of commercial PU can be further enhanced.

## Figures and Tables

**Table 1:** Synthesis recipes for O-I hybrid solutions.

Solution	Ingredients			
	AFAP	APTES	Ethanol	HCl (0.1 M)
ATS 1-1	5 g	5 g	40 g	1.54 g
ATS 1-2	5 g	10 g	40 g	2.77 g
ATS 2-1	5 g	2.5 g	40 g	0.93 g

**Table 2:** Preparation recipes for O-I hybrid NPs dispersed in BG.

No	Sample	Ingredients	
		ATS	BG
1	ATS (1-1)–BG (30)	50 g	30 g
2	ATS (2-1)–BG (30)	50 g	30 g
3	ATS (1-2)–BG (30)	50 g	30 g
4	ATS (1-2)–BG (40)	50 g	40 g
5	ATS (1-2)–BG (50)	50 g	50 g

**Table 3:** Ingredients of pristine SB/EB PUs.

No	Ingredients	Resin		Hardener	
		wt%	g	wt%	g
1	SB/EB polyol	40.60%	2.029	80.70%	2.738
2	Moisture absorbent	5.04%	0.252	-	
3	Filler	54.40%	2.72	19.40%	0.66
	Total weight	5.001		3.398	
	Weight ratio	1: 0.68			

**Table 4:** Ingredients of SB/EB PUs mixed with different amount of ATS–BG.

No	Ingredient		Resin (g)			Hardener (g)
			1 wt%	3 wt%	5 wt%	
1	SB/EB polyol containing ATS–BG	SB/EB polyol	2.029	2.029	2.029	2.738
		ATS ^(^*^)^–BG ^(^**^)^	0.02	0.061	0.101	0
2	Moisture absorbent		0.252	0.252	0.252	0
3	Filler		2.72	2.72	2.72	0.66
Total weight (g)/			5.021	5.062	5.102	3.398
Mass difference (%) compared to pristine PU			0.24%	0.72%	1.19%	

^(^*^)^ Mass ratio of AFAP to APTES: (1-2), (1-1), and (2-1). ^(^**^)^ Amount of BG in solvent exchange process: 30, 40, and 50.

**Table 5:** Experimental variables and their coded levels for RSM–CCD.

Independent Variable	Symbol	Unit	Range and Level of Coded Variables				
			−α (−1.667)	Coded Low (−1)	Mean (0)	Coded High (+1)	+α (+1.682)
AFAP	X_1_	g	2.58	3.6	5.1	6.6	7.62
APTES	X_2_	g	5.56	7.2	9.6	12	13.64
BG	X_3_	g	9.77	20	35	50	60.23
Mixing ratio	X_4_	%	0	1	3	5	6.36

**Table 6:** Properties of O-I hybrid nanoscopic fillers.

O-I Hybrid Solutions		Particle Size (nm) ^a^	Moisture Content (wt%)	Assigned Structures Si_x_O_2x+1_(C_2_H_5_)_x+2_(C_3_H_6_NH_2_)_x_^b^	DOC ^c^
1	ATS (1-1)	11.66	-	Si3 → Si6	97.19%
2	ATS (2-1)	32.86	-	Si3 → Si6	93.59%
3	ATS (1-2)	14.32	-	Si3 → Si9	98.74%
4	ATS (1-1)–BG (30)	30.78	0.04	-	94.86%
5	ATS (2-1)–BG (30)	38.51	0.08	-	90.88%
6	ATS (1-2)–BG (30)	30.86	0.06	-	97.06%
7	ATS (1-2)–BG (40)	25.10	0.07	-	97.02%
8	ATS (1-2)–BG (50)	22.80	0.08	-	97.59%

^a^ Size of O-I hybrid NPs dispersed in ethanol. ^b^ Chemical structures assigned for polysiloxane compounds after hydrolysis–condensation. ^c^ DOC is the abbreviation for degree of condensation.

**Table 7:** Properties of O-I hybrid nanoscopic fillers.

Sample	*A*_1733_ *	*A*_1710_ **	*R* ***	*DPS*	*DPM*
Pristine EB PU	7.26	2.02	3.59	0.78	0.22
ATS EB PU	7.2	1.68	4.29	0.81	0.19
Pristine SB PU	9.28	1.87	4.96	0.83	0.17
ATS SB PU	7.62	1.3	5.86	0.85	0.15

A: absorption intensity calculated as the area of Gaussian multipeak fitting [absorbance * wavenumber]; A_1733_ *: absorption intensity of free carbonyl. A_1710_ **: absorption intensity of hydrogen-bonded carbonyl. R = A_1710_/A_1733_ ***: carbonyl hydrogen bonding index.

**Table 8:** ANOVA for adhesion strength (MPa).

Source	Sum of Squares	df	Mean Square	F-Value	*p*-Value	Contribution (%)
Model	33.35	14	2.38	13.55	<0.0001 (s)	92.66%
A-AFAP	2.66	1	2.66	15.14	0.0014	7.39%
B-APTES	2.11	1	2.11	12.01	0.0035	5.86%
C-BG	0.8358	1	0.8358	4.75	0.0456	2.32%
D-Mixing amount	0.1600	1	0.1600	0.9099	0.3553	0.44%
AB	0.8202	1	0.8202	4.66	0.0474	2.28%
AC	0.4078	1	0.4078	2.32	0.1486	1.13%
AD	0.0098	1	0.0098	0.0555	0.8169	0.03%
BC	2.57	1	2.57	14.63	0.0017	7.14%
BD	0.1233	1	0.1233	0.7013	0.4155	0.34%
CD	0.0022	1	0.0022	0.0123	0.9131	0.01%
A^2^	1.09	1	1.09	6.22	0.0248	3.03%
B^2^	5.90	1	5.90	33.55	<0.0001	16.39%
C^2^	13.97	1	13.97	79.41	<0.0001	38.82%
D^2^	1.13	1	1.13	6.44	0.0228	3.14%
Residual	2.64	15	0.1759			7.34%
Lack of Fit	2.04	10	0.2042	1.71	0.2871 (ns)	
Pure Error	0.5958	5	0.1192			
Cor Total	35.99	29				100%

**Table 9:** Statistical indicators and model verification.

Std. Dev.	0.4162	R^2^	0.9267
Mean	3.26	Adjusted R^2^	0.8583
C.V. %	12.75	Predicted R^2^	0.6597
Adeq. Precision			10.2524

**Table 10:** Optimal processing conditions and model validation of adhesion strength from numerical optimization.

Parameters	AFAP (g)	APTES (g)	BG (g)	ATS Mixing Ratio (%)	Adhesion Strength Mpa	
					Predicted	Experimental
Optimum conditions	4.36	10.25	37.16	3.6	4.94	4.88

## Data Availability

The data presented in this study are available on request from the corresponding author.

## Supplementary Materials

The following supporting information can be downloaded at https://www.mdpi.com/article/10.3390/polym16192816/s1, Table S1: CCD matrix of 4 variables along with experimental and predicted responses.
