# Supplementary material for: Organic–Inorganic Hybrid Nanoparticles for Enhancing Adhesion of 2K Polyurethane to Steel and Their Performance Optimization Using Response Surface Methodology
Source: Polymers (Basel). 2024 Oct 4;16(19):2816. doi: 10.3390/polym16192816 (PMC11478698; doi:10.3390/polym16192816)
Supplement: Supplementary file 1 [file polymers-16-02816-s001.zip › polymers-3208974-supplementary.pdf]

Supplementary Materials

Organic–Inorganic Hybrid Nanoparticles for Enhancing Adhesion of 2K Polyurethane to Steel and Their Performance Optimization Using Response Surface Methodology

Thu Thuy Duong [1,†], Manh Linh Le [1,2,†], Changhoon Lee [1] and Juyoung Kim [1,*]

[1] Nanocomposite Structure Polymer Lab, Department of Advanced Materials Engineering, Kangwon
National University, Samcheok 25913, Republic of Korea; thuy.dgth@gmail.com (T.T.D.); haryml07@gmail.com (M.L.L.); (chlee@dankook.ac.kr)

[2] VN-UK Institute for Research and Executive Education, The University of Danang, Danang 550000, Vietnam

* Correspondence: juyoungk@kangwon.ac.kr

† These authors contributed equally to this work.

Table S1: CCD matrix of 4 variables along with experimental and predicted responses

Run	AFAP	APTES	BG	Mixing ratio	Response Y
1	5.1	5.5632	35	3	2.1232
2	5.1	9.6	35	3	5.19
3	2.577	9.6	35	3	4.26
4	6.6	7.2	20	1	1.5104
5	6.6	12	20	5	2.068
6	6.6	12	50	5	3.7736
7	5.1	9.6	35	6.364	3.592
8	3.6	7.2	50	5	2.852
9	6.6	7.2	20	5	2.08
10	5.1	9.6	35	0	4.484
11	3.6	7.2	50	1	2.708
12	7.623	9.6	35	3	3.68
13	5.1	13.6368	35	3	3.88
14	6.6	7.2	50	5	2.08
15	3.6	7.2	20	5	3.44
16	5.1	9.6	35	3	4.75
17	3.6	12	50	5	4.016
18	3.6	7.2	20	1	3.6123
19	3.6	12	20	5	3.2008
20	5.1	9.6	35	3	4.288
21	5.1	9.6	60.23	3	2.1456
22	6.6	12	20	1	2.0392

23	3.6	12	20	1	2.0312
24	6.6	12	50	1	3.326
25	5.1	9.6	9.77	3	2.025
26	5.1	9.6	35	3	4.616
27	5.1	9.6	35	3	5.181
28	6.6	7.2	50	1	1.821
29	5.1	9.6	35	3	4.82
30	3.6	12	50	1	5.731
